# Assessment of inter-examiner agreement and variability in the manual classification of auditory brainstem response

**DOI:** 10.1186/1475-925X-11-86

**Published:** 2012-11-22

**Authors:** Kheline FP Naves, Adriano A Pereira, Slawomir J Nasuto, Ieda PC Russo, Adriano O Andrade

**Affiliations:** 1 Laboratory of Biomedical Engineering, Faculty of Electrical Engineering, Federal University of Uberlândia, Av. João Naves de Ávila, 2160 - Bl. 3N - Campus Santa Mônica, Uberlândia, MG 38400-902, Brazil; 2 University of Reading, School of Systems Engineering, Cybernetic Intelligence Research Group, Reading, UK; 3 (in memorian) Catholic University of São Paulo, São Paulo, Brazil

**Keywords:** Auditory brainstem response, ABR classification, Inter-examiner variability

## Abstract

**Background:**

The analysis of the Auditory Brainstem Response (ABR) is of fundamental importance to the investigation of the auditory system behaviour, though its interpretation has a subjective nature because of the manual process employed in its study and the clinical experience required for its analysis. When analysing the ABR, clinicians are often interested in the identification of ABR signal components referred to as Jewett waves. In particular, the detection and study of the time when these waves occur (i.e., the wave latency) is a practical tool for the diagnosis of disorders affecting the auditory system. Significant differences in inter-examiner results may lead to completely distinct clinical interpretations of the state of the auditory system. In this context, the aim of this research was to evaluate the inter-examiner agreement and variability in the manual classification of ABR.

**Methods:**

A total of 160 ABR data samples were collected, for four different stimulus intensity (80dBHL, 60dBHL, 40dBHL and 20dBHL), from 10 normal-hearing subjects (5 men and 5 women, from 20 to 52 years). Four examiners with expertise in the manual classification of ABR components participated in the study. The Bland-Altman statistical method was employed for the assessment of inter-examiner agreement and variability. The mean, standard deviation and error for the bias, which is the difference between examiners’ annotations, were estimated for each pair of examiners. Scatter plots and histograms were employed for data visualization and analysis.

**Results:**

In most comparisons the differences between examiner’s annotations were below 0.1 ms, which is clinically acceptable. In four cases, it was found a large error and standard deviation (>0.1 ms) that indicate the presence of outliers and thus, discrepancies between examiners.

**Conclusions:**

Our results quantify the inter-examiner agreement and variability of the manual analysis of ABR data, and they also allows for the determination of different patterns of manual ABR analysis.

## Background

The study of the Auditory Brainstem Response (ABR) is an important tool for the evaluation of the auditory capacity and plasticity, as well as for the investigation of the integrity of the structures involved in the transmission of electrical impulses through the auditory system [1-3]. The classical process of analysis of the ABR consists in the identification of relevant temporal and morphological features of the Jewett waves, which are basic components of the ABR. The waves I, III and V are characterized by presenting the most evident positive peaks in the whole signal, and they are usually employed for the evaluation of the integrity of the auditory pathway [4-6].

When the objective of the ABR exam is the investigation of electro-physiological thresholds, the wave V is the most relevant, as it remains more evident in the signal even under low power intensity (e.g., 20 dB) [3]. Currently, ABR analysis can be employed in distinct contexts. For instance, it can be used for the determination of electro-physiological thresholds in children, diagnosis of neural dysfunctions [1,7], intra-operative monitoring [8], cardiac surgery, staging of coma, detection of degenerative diseases that produce hearing impairment, and in the diagnosis of auditory disorders that cannot be identified by tonal audiometry (e.g., in some motor deficiencies) [9].

The most common use of ABR analysis in clinical practice is the diagnosis of early hearing loss, particularly in newborns and children. According to the World Health Organization (WHO), 1.4 million children worldwide suffer from hearing problems. Olusanya *et al*. [10] reported that 855 babies are born every day in developing countries with hearing loss with little expectation of being diagnosed. A late diagnosis may hamper the cognitive development of patients, language skills, consequently resulting in delay of the learning and emotional processes [11,12]. Another relevant application of ABR analysis is in the identification of diseases in the auditory nerve, such as tumor (schwannoma), neuropathy, dys-synchrony and degenerative diseases affecting the brainstem.

In most clinical situations, the ABR waves are identified through a manual assessment. The process of identification of the ABR components is dependent upon many variables, such as the employed experimental protocol, the clinical conditions of the subject and more importantly, on the previous experience of the examiner. The manual analysis of the ABR yields inconsistency in the results obtained by distinct examiners [13-15]. This makes the process of identification of the Jewett waves prone to error and can contribute to the erroneous diagnosis of some diseases. The consequences of a non-precise diagnosis are numerous, for instance, leading to inadequate treatment, or even delaying discovery of a serious illness.

In this context, given the importance of the ABR analysis and the subjective nature of its interpretation, the main objective of this study was to evaluate the inter-examiner agreement and variability in the manual classification of ABR. The examiners focused their analysis on classical features (i.e., temporal and morphological) manually extracted from the signal, as it is practiced in the clinical routine.

The results of this study quantify the variability found in the responses given by the examiners. Such results can be useful for highlighting the necessity of continuing training and standardization of procedures used for the interpretation of the ABR in the clinical practice. In the future, they can also be employed in the development of more accurate intelligent algorithms used for the automatic detection of the ABR waves.

## Methods

In total, ten subjects (five men and five women), with mean age of 36 years (minimum=20 and maximum=52), participated in the experiments. Subjects were selected based on their performance in standard exams that verify the integrity of the auditory system. The following exams were applied: otoscopy, pure tone audiometry and speech audiometry (WRS–Word Recognition Score and SRT – Speech Recognition Threshold) for the confirmation of the normal hearing thresholds. The audiometer model AC40 (Interacoustics, USA), duly calibrated according to recent international technical norms was employed. Pure tone thresholds were considered as normal from 0 to 25dBHL (HearingLevel), in the frequencies of 250Hz, 500Hz, 1kHz, 2kHz, 3 kHz, 4kHz, 6kHz and 8kHz. Prior to data collection, the subjects signed a Consent Form approved by the Ethical Committee of the Federal University of Uberlândia, Uberlândia, Brazil (ProjectID:160/06). Four examiners (E1, E2, E3 and E4) with expertise in audiology participated in this study. All of them had theoretical and practical experience in the detection and analysis of ABR as shown in Table 1.

**Table 1:** Experience in years for each examiner

***Examiner***	***Experience (years)***	
	**Audiology**	**ABR analysis**
E1	11	9
E2	6	6
E3	9	3
E4	15	11

### Data collection

ABR data were collected by means of the commercial amplifier *Bio-logic’s Evoked Potential System* (EP), from Bio-Logic, USA. Prior to the positioning of electrodes on the scalp of the subject, the skin was properly cleansed and abraded. The electrodes were positioned according to the International 10–20 System proposed by Jasper in 1958 [6], being M1 (mastoid right) and M2 (mastoid left), Cz (active) and Fz (ground). Two differential channels of information were recorded. Channel 1 (M1-Cz), representing information detected from the right ear and Channel 2 (M2-Cz) from the left ear.

The signals were collected at a sample rate of 37,101 Hz, meaning that the time interval between two consecutive samples was of 0.027 ms. Each signal, resulting from an auditory stimulus, lasted 13.824 ms (or 512 samples). In this study we work with the averaged ABR, which is obtained by averaging 2000 ABR samples. This process can be seen as a filter that reduces background activity and highlights the signal of interest. The auditory stimulus (clicks) was used for the 80, 60, 40 and 20 dBHL power intensities for each ear. The stimulus rate was set to 21 cycles/s, as commonly used in clinical practice. The auditory stimulus used was the Click in the following intensities 80, 60, 40 and 20 dBHL for each ear. This procedure was repeated twice, resulting in 160ABR samples.

### Data analysis

The examiners followed their individual criteria and professional experience, and their analyses consisted in the manual classification of waves I, II, III, IV and V. The results of this classification were the identification of the peak of the wave (amplitude) and its corresponding time of occurrence. Based on these results it was possible to estimate the inter-examiner agreement and variability by using the Bland-Altman statistical method. This method is a tool that has been cited on more than 11,500 research studies [16], highlighting its relevance in medical research.

The application of the Bland-Altman analysis is straightforward: (i) given two random variables ***x*** and ***y***, first, a scatter plot relating the mean (***x*+y**)/2, in the *x* axis, and the bias (i.e., ***x-y***) in the *y* axis, is generated; (ii) the hypothesis of the bias is equal to zero is assessed by means of a paired *t*-test (*p<0.05*); (iii) the 95% agreement confidence interval is included in the plot generated in (i), and it can be estimated as *bias*±1.96*std*, where *std* is the standard deviation of the bias; (iv) finally, the error as defined in (1) is estimated, where *Err* is the mean absolute error, *N* the dimension of the random vectors ***x*** and ***y***.

(1) $$Err=\frac{\left|\Sigma_{i=1}^{N}\left(x_{i}-y_{i}\right)-\left(\frac{x-y}{2}\right)\right|}{N}$$

## Results

### Data consistency analysis

**Figure 1:**
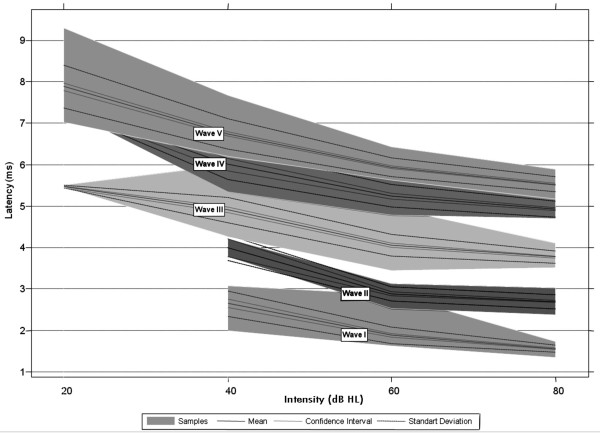
**Latency values obtained for each Jewett wave as function of the intensity (dBHL).** The shaded areas are bounded by the minimum and maximum values of latency found for each wave. The standard deviation, the central tendency and its 95% confidence interval are also presented.

The first step in signal analysis is the visual inspection of the collected data. This can help the detection of outliers, patterns and possible inconsistencies in the data set. Figure 1 shows a graph of the intensity (in dBHL) versus the latency (in ms) provided by the four examiners, for all subjects, and for waves I, II, III, IV and V.

The results include the analysis of 160 ABR samples. In the graph the shaded areas represent the area limited by the minimum and maximum latency values obtained for the analysis for each wave and intensity. In addition, the standard deviation of the samples is presented together with a central tendency (i.e., the mean) and its 95% confidence interval estimated by means of Bootstrap [17].

The visual inspection of the graph reveals that the latency increases as the intensity decreases. This behavior is in accordance with findings reported in literature, which discusses the differences in the ABR patterns as function of the intensity [1,18-20]. Another relevant observation is that at the 80 dBHL intensity, the ABR signal has a relatively high signal-to-noise ratio, which allows for a more precise evaluation of the waves, as they are more evident. For this reason, at the high intensity the latency is an important discriminatory feature of the Jewett waves. Note in the graph that at this intensity there is no overlap between the shaded areas and the central tendencies of the waves.

However, as we decrease the intensity, the visual detection of some waves is impaired. For instance, the examiners could not visually detect the presence of waves I and II at the 20 dBHL intensity. Wave III is more evident in the intensities of 80, 60 and 40 dBHL. In the 20 dBHL intensity the number of detections was significantly smaller. Waves IV and V remain evident for all intensities, but they tend to overlap at the 20 dBHL, as the detection of waves IV and V gets more complex (because the signal amplitude for this intensity tends to decrease). The number of detections is significantly lower at low intensity. This happens because of the way the neurons are activated by low stimulus intensity.

In general waves I, II and III are less evident at lower intensity, different from waves IV and V, which are evident even at low intensity, being therefore employed in auditory threshold detection studies.

The experimental results given in Figure 1 are in accordance with those found in literature [1,19,21,22], showing, therefore, the consistency of our data set and the visual detection of the Jewett waves executed by the examiners.

### Inter-examiner agreement and variability analysis

Table 2 presents the results of the application of the Bland-Altman analysis for each pair of examiners and type of wave. In this table the mean and standard deviation of the bias are given, together with the error (*Err*) defined in (1).

**Table 2:** The mean, standard deviation and error (see (1)) for the bias and each pair of examiners is presented

		**Wave**				
**Pair**	**Bias (x-y)**	**I**	**II**	**III**	**IV**	**V**
	*mean (ms)*	-0.018	-0.030	-0.039	-0.039	0.055
**E1E2**	*std (ms)*	0.055	0.045	0.082	0.086	0.086
	*Err (ms)*	0.003	0.002	0.007	0.007	0.007
	*mean (ms)*	0.008^*^	-0.009^*^	-0.023	-0.027^*^	-0.080
**E1E3**	*std (ms)*	0.046	0.039	0.079	0.107^§^	0.088
	*Err (ms)*	0.002	0.002	0.006	0.011^§^	0.008
	*mean (ms)*	-0.001^*^	-0.028	-0.027	-0.044	-0.030
**E1E4**	*std (ms)*	0.040	0.044	0.078	0.087	0.073
	*Err (ms)*	0.002	0.002	0.006	0.007	0.005
	*mean (ms)*	0.028	0.020	0.019	0.000^*^	-0.026
**E2E3**	*std (ms)*	0.051	0.049	0.073	0.110^§^	0.104^§^
	*Err (ms)*	0.003	0.002	0.005	0.012^§^	0.011^§^
	*mean (ms)*	0.012^*^	0.004^*^	0.012^*^	0.008^*^	0.023
**E2E4**	*std (ms)*	0.047	0.056	0.080	0.073	0.081
	*Err (ms)*	0.002	0.003	0.006	0.005	0.007
	*mean (ms)*	0.000^*^	-0.018	-0.007^*^	0.000^*^	0.050
**E3E4**	*std (ms)*	0.118^§^	0.035	0.035	0.084	0.070
	*Err (ms)*	0.014^§^	0.001	0.001	0.007	0.005

Statistical significant results that confirmed the hypothesis of null bias are highlighted with *. The unit of the data in the table is *ms*. Cells marked with ‘^§^’ are the results that presented the largest error and standard deviation.

**Figure 2:**
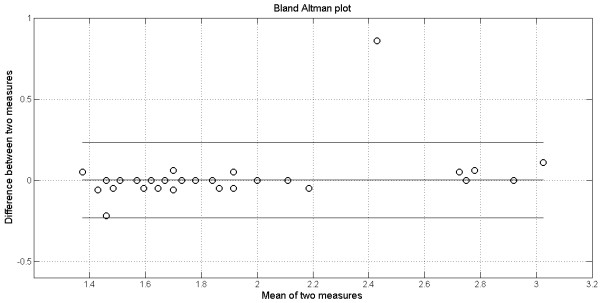
**Bland-Altman plot for annotation agreement between examiners 3 and 4, for wave I.** The bias, its 95% confidence interval and mean are given.

Figure 2 depicts the plot obtained when applying the Bland-Altman method for the analysis in which the error is maximal (0.014 – see Table 1). Results concerning examiners 3 and 4, and wave I, are presented. The bias and its confidence interval are given. As one can notice most annotations agree because they are in the confidence interval region, however there is an outlier that justifies the large error.

**Figure 3:**
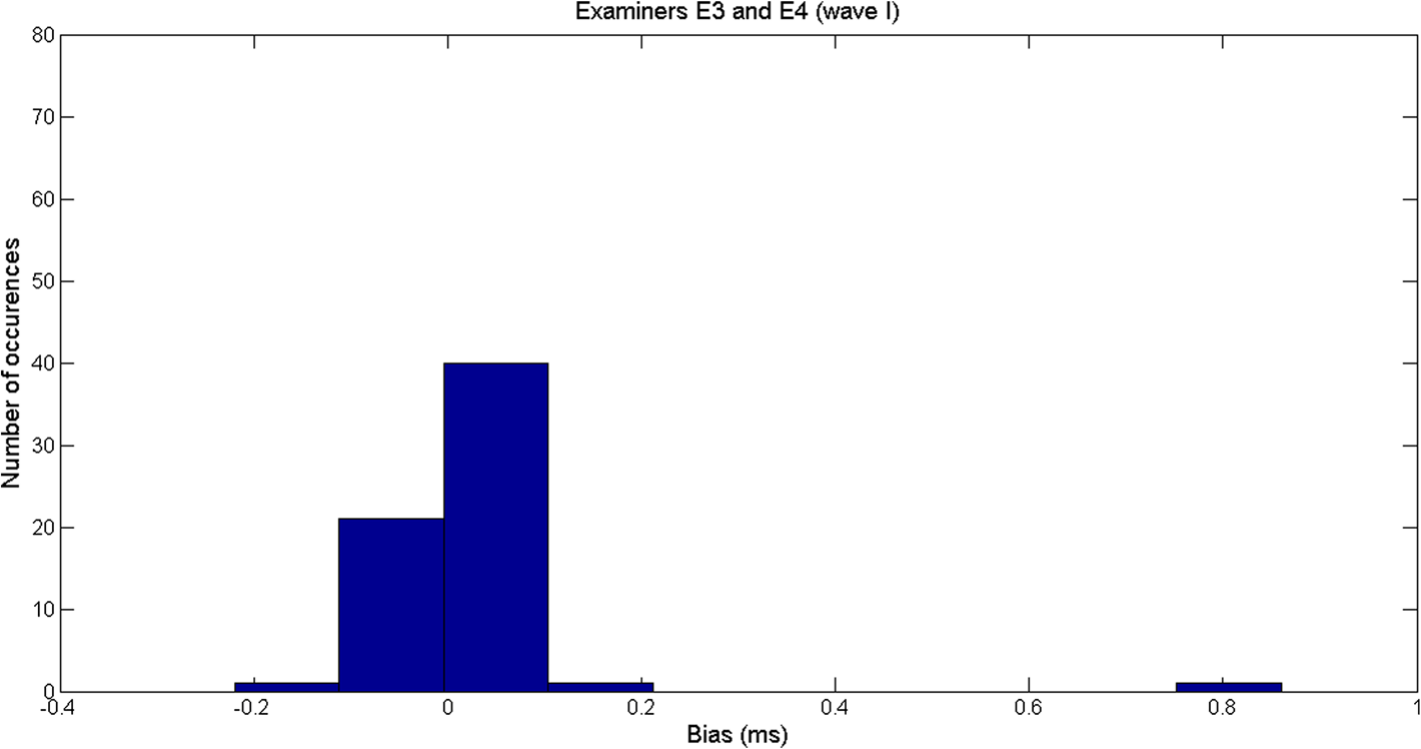
Histogram of the bias for the examiners E3 and E4 for the wave I.

Figure 3 shows the histogram of the bias (samples in Figure 2). Most differences lie between −0.1 ms and 0.1 ms, which are acceptable in clinical analysis. The outlier can be easily detected in the histogram.

**Figure 4:**
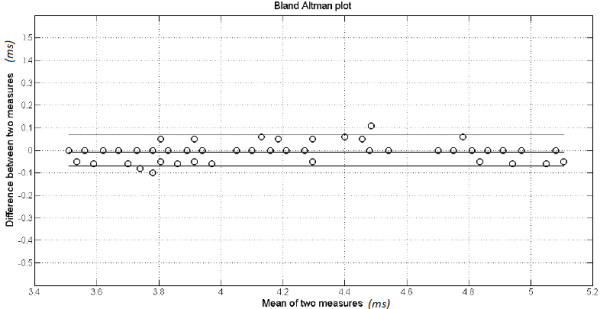
**Bland-Altman plot for annotation agreement between examiners 3 and 4, for wave III.** The bias, its confidence interval and mean are given.

Figure 4 shows the Bland-Altman plot for examiners 3 and 4, and wave III. This was the smallest error found between examiners. Most differences are less than 0.1 ms.

**Figure 5:**
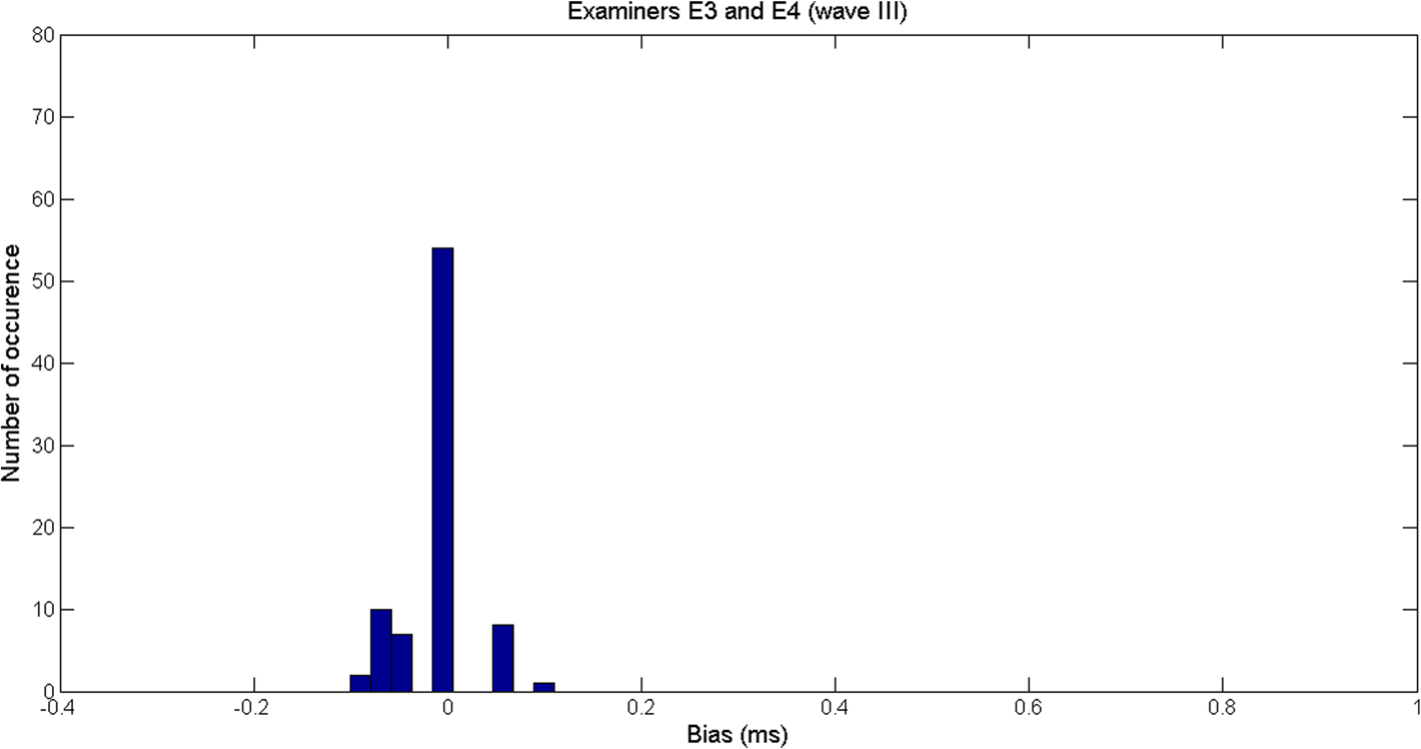
Histogram of the bias for the examiners E3 and E4 for the wave III.

Figure 5 shows the histogram of the bias of annotations from examiners 3 and 4, for wave III. Most differences are around zero, illustrating thus a considerable agreement between the examiners.

## Discussion and conclusion

The main objective of this study was to investigate the inter-examiner agreement and variability in the manual analysis of ABR provided by four seasoned examiners.

The motivation of this research comes from our own clinical experience that have shown that subjectivity and lack of standards in the interpretation of ABR is common and can lead to erroneous and inaccurate diagnosis of disorders that affect the auditory system. This subjectivity is also reported in many published research works [13,23].

The first stage of our analysis was to verify whether the latency values obtained by the examiners were compatible with those reported in the literature. The results presented in Figure 1 depict all information provided by the examiners.

They are consistent with patterns described in other studies. For the intensity of 80 dBHL we obtained the following mean values for the Jewett waves: 1.56 ms (wave I), 3.77 ms (wave III) and 5.53 ms (wave V). Antonelli [22] reported that the normal average values of latency in the 100 dBSPL (Sound Pressure Level) intensity for waves I, III and V are respectively equal to 1.54 ms, 3.73 ms and 5.52 ms. Hernandez [19] evaluated the behavior of waves generated at different power intensities. In the intensities of 90, 70, 50, 30 and 10 dBHL the wave V was always detected and the average latency values were 1.49 ms, 3.73 ms and 5.53 ms for the waves I, III and V, respectively. These results indicate the coherence in the manual analysis provided by the examiners in this research.

Another problem we had to face in our analysis was in the establishment of acceptable threshold levels for the variation of the latency of Jewett waves. There is some disagreement in literature, as some authors report a variation of 0.1 ms as acceptable, whereas others report 0.2 ms [1,5,13,21,23]. In addition, some studies concerning the development of automatic systems for the detection of Jewett waves have considered values of latency between 0.1 ms and 0.2 ms as acceptable for the validation of these systems [24-27].

In our study, even though the application of the statistical paired *t*-test did not confirm a null bias for all pair of examiners and waves, the analysis of the mean, standard deviation and error showed that the discrepancies between examiners are below those considered clinically acceptable (see Figures 3 and 5).

The analysis of the error is important because it allows for the detection of outliers. For instance, the large error (0.014) found for examiners E3 and E4 in comparison to other results, reveals the presence of an outlier (0.8 ms), which is not clinically acceptable (see Figures 2 and 3). Large errors like this may lead to misdiagnosis that can have serious consequences to patients.

Besides the error it is also important to assess the standard deviation, as it is a measure of data variability. For instance, for wave IV, examiners E1 and E3, and also for examiners E2 and E3, the standard deviation is relatively large (>0.1 ms, see Table 2) which is also an indicative of discrepancies between examiners.

In general, when considering the variables involved in the process of ABR analysis, such as subjectivity and the number of years of experience of examiners, our results showed that there is a consistency between the annotations provided by the examiners. In most comparisons the variability found in the results was not clinically relevant since they are below 0.1 ms, though a more detailed study of the cases that presented large error and standard deviation suggested relevant discrepancies (e.g., outliers) between examiners.

A relevant finding of the study was that the experience in years in ABR analysis was not a determinant criterion in the success of the agreement between examiners. In our investigations examiners with different experience showed compatible results as can be seen in Tables 1 and 2. However, the largest disagreements between examiners’ annotations (see Table 1, cells marked with ‘^§^’), had the participation of examiner E3, who is the less seasoned examiner. This may suggest that this examiner needs further training in ABR analysis.

Occasional large differences between examiners may happen due to many factors: (i) misdetection of the peak of the ABR wave during the manual process of data analysis; (ii) lack of standardization during the process of peak identification; and (iii) introduction of error during the annotation procedure which involves transferring data from the computer screen to a spreadsheet.

The main contributions of this research were: (i) determination of patterns of manual annotations, for different stimulus intensity and waves, for a specific group of examiners; (ii) the proposal of a method capable of detecting examiners that have different patterns of ABR analysis; (iii) the possibility of applying the results to the development and evaluation of automatic systems for detecting ABR waves.

## Competing interests

The authors declare that they have no competing interests.

## Authors' contributions

KN: Participated in the conception of the study, in data collection, in data analysis, and drafted the manuscript. AOA: Participated in the conception of the study, in data collection, in data analysis, and drafted the manuscript. SJ: Participated in the data analysis. IP: Participated in this study with her knowledge in practical clinical and in the definition of experimental protocols. AA: Participated in the data analysis. All authors read and approved the final manuscript.
